# Article of Significant Interest in This Issue

**DOI:** 10.1128/iai.00237-25

**Published:** 2025-05-13

## NEW INSIGHTS INTO THE *HELICOBACTER PYLORI* CAG TYPE IV SECRETION SYSTEM

The *Helicobacter pylori* Cag type IV secretion system (T4SS) is a macromolecular machine that delivers protein and non-protein substrates into gastric cells. At least 17 *H*. *pylori* proteins are required for Cag T4SS activity, including several that are absent from prototypical T4SSs in other species. Protein-protein interactions among putative components of the T4SS are incompletely defined. Bryant et al. (e00493-24) identified four species-specific components of the Cag T4SS that co-purify with the T4SS outer membrane core complex and are required for T4SS activity. These findings improve our understanding of a T4SS that has a key role in gastric cancer pathogenesis.

